# Clinical Profile, Indications, and Maternal Outcomes of Obstetric Intensive Care Unit (ICU) Admissions: A Retrospective Study With Emphasis on Critical Care Interventions

**DOI:** 10.7759/cureus.117574

**Published:** 2026-10-07

**Authors:** Saurabh Chiragbhai Madhukar, Kalyani Anilkumar Jayachithra, Reshmabnu Rajubhai Zakhara, Ritesh Mate, Divya Pula, Harsh V Salankar

**Affiliations:** 1 Obstetrics and Gynaecology, GCS Medical College, Hospital & Research Centre, Ahmedabad, IND; 2 Surgery, Georgian International University, Idukki, IND; 3 Emergency Medicine, Tianjin Medical University, Tianjin, CHN; 4 Cardiovascular and Thoracic Surgery, Government Medical College and Hospital, Nagpur, IND; 5 General Medicine, NRI Academy of Sciences, Guntur, IND; 6 Pharmacology, N.K.P. Salve Institute of Medical Sciences, Nagpur, IND

**Keywords:** hypertensive disorders, maternal mortality, maternal outcomes, obstetric hemorrhage, obstetric icu

## Abstract

Background

Maternal morbidity and mortality continue to pose considerable problems to global health systems, especially in developing countries like India. The monitoring of the number of patients admitted to obstetric intensive care units (ICU) is an important measure of the incidence of severe maternal morbidity and the quality of emergency obstetric care available to patients.

Objective

To improve maternal health care services, there must be an understanding of the clinical characteristics, reasons for admission, and outcomes of these patients.

Methods

A retrospective observational study was conducted in the obstetric ICU of a tertiary care teaching hospital from January 2025 through December 2025. A total of 120 obstetric patients (pregnant women and women within 42 days postpartum) admitted to the ICU were included. Information regarding patients' demographic characteristics, obstetric profile, reasons for ICU admission, critical care interventions performed, and maternal outcomes was obtained from medical records. The collected data were analyzed using SPSS version 25.0 (IBM Corporation, Armonk, USA).

Results

A total of 120 obstetric patients were admitted to the intensive care unit. Hypertensive disorders were the most common indication for ICU admission (42; 35%), followed by obstetric hemorrhage (36; 30%) and sepsis (18; 15%). Blood transfusion was the most frequently provided critical care intervention (84; 70%), followed by mechanical ventilation (48; 40%), vasopressor support (30; 25%), and dialysis/renal replacement therapy (6; 5%). Overall, 102 (85%) patients recovered and were discharged, while 18 (15%) patients died. Delayed referral, multi-organ dysfunction, and requirement for mechanical ventilation were significantly associated with maternal mortality (all p < 0.001).

Conclusion

Hypertensive disorders and obstetric hemorrhage were the leading indications for obstetric ICU admission. Delayed referral, multi-organ dysfunction, and requirement for mechanical ventilation were significantly associated with maternal mortality. Timely referral and access to multidisciplinary critical care remain important in the management of critically ill obstetric patients.

## Introduction

Maternal morbidity and mortality continue to be important public health concerns, particularly in low- and middle-income countries such as India [1]. Although maternal mortality has declined substantially over the past several decades with improvements in obstetric care and healthcare delivery, pregnant and postpartum women may still develop severe and potentially life-threatening complications during pregnancy, childbirth, or the postpartum period [2]. These complications may require admission to an intensive care unit (ICU) for advanced monitoring, organ support, and multidisciplinary management [3].

Obstetric ICU admissions provide an important measure of severe maternal morbidity and reflect both the burden of critical obstetric illness and the capacity of a healthcare system to provide timely emergency and critical care [4]. The common reasons for admission include hypertensive disorders of pregnancy, particularly preeclampsia and eclampsia; obstetric hemorrhage; sepsis; and cardiac or other medical conditions complicating pregnancy [5]. When these conditions are not recognized and treated promptly, they may progress to severe organ dysfunction and maternal death [6].

Maternal near miss (MNM) refers to a woman who nearly died but survived a complication occurring during pregnancy, childbirth, or within 42 days of termination of pregnancy [7]. Evaluation of maternal near-miss cases and critically ill obstetric patients can provide useful information regarding gaps in antenatal care, referral pathways, and emergency management. In particular, delays in recognizing severe illness, reaching an appropriate healthcare facility, or receiving definitive treatment may contribute to adverse maternal outcomes [7,8].

Recent advances in critical care have improved the management of critically ill obstetric patients. Mechanical ventilation, vasopressor support, blood and blood-component transfusion, and renal replacement therapy are important supportive interventions used when severe organ dysfunction develops [8]. However, outcomes may vary according to the severity of illness at presentation, timing of referral, availability of resources, and access to trained multidisciplinary critical care teams [9]. In resource-limited settings, inadequate access to emergency obstetric services, delayed referral, and insufficient antenatal care may further increase the risk of adverse maternal outcomes [10].

Pregnancy is associated with substantial physiological changes involving the cardiovascular, respiratory, and hematological systems, which can complicate the assessment and management of critically ill pregnant and postpartum women [11]. Therefore, effective management of obstetric ICU patients requires coordination between obstetricians, intensivists, anesthesiologists, nursing staff, and other relevant healthcare professionals [12].

Despite the importance of obstetric critical care, understanding the characteristics of women requiring ICU admission, the clinical conditions leading to admission, the critical care interventions provided, and the subsequent maternal outcomes remains essential for identifying areas where obstetric and critical care services can be strengthened.

Therefore, the primary objective of this retrospective study was to describe the demographic and clinical characteristics and indications for ICU admission among obstetric patients admitted to a tertiary care hospital. The secondary objectives were to describe the critical care interventions provided and to evaluate maternal outcomes, including recovery, discharge, and mortality, as well as the association of selected clinical factors with maternal mortality.

## Materials and methods

Study design and setting

A retrospective observational study was conducted in the obstetric ICU of a tertiary care teaching hospital from January 2025 to December 2025. The hospital serves as a referral centre for the surrounding rural and semi-urban population and provides comprehensive obstetric and critical care services, including emergency obstetric care and multidisciplinary ICU management. The study included obstetric patients admitted to the ICU during the study period. Obstetric patients were defined as women who were pregnant at the time of ICU admission or who were within 42 days of delivery or abortion.

Participant selection

During the study period, records of 250 patients admitted to the ICU were screened for eligibility. Of these, 126 patients did not meet the predefined inclusion criteria and were excluded. Four additional patients were excluded because of incomplete medical records. Thus, a total of 120 patients met the eligibility criteria and were included in the final analysis. The study used complete enumeration of all eligible obstetric ICU admissions during the study period rather than selecting a predetermined sample from the eligible population.

Inclusion criteria

Patients were included if they fulfilled all of the following criteria: 1) pregnant women admitted to the ICU during the study period, irrespective of gestational age; 2) women admitted to the ICU within 42 days after delivery or abortion; 3) patients requiring critical care because of an obstetric complication or a medical condition associated with pregnancy; and 4) availability of sufficient medical records to obtain the predefined study variables and outcomes.

Exclusion criteria

Patients were excluded if they had: 1) ICU admission for a non-obstetric gynecological indication; 2) incomplete or unavailable medical records that prevented assessment of the predefined study variables or outcomes; or 3) records that did not contain sufficient information to establish eligibility for the study.

Booking status was recorded from the antenatal records. A booked patient was defined as a woman who had received documented antenatal care at the study hospital or an established healthcare facility before the current admission. An unbooked patient was defined as a woman without documented regular antenatal care before the ICU admission. Data were collected using a structured data extraction form from hospital medical records, ICU admission registers, case records, and discharge summaries. Demographic variables included age, parity, and booking status. Obstetric variables included gestational age at admission, antenatal or postnatal status, obstetric complications, and mode of delivery. Gestational age was recorded as a descriptive clinical variable and was not used as an exclusion criterion.

Clinical variables included the primary indication for ICU admission, associated comorbidities, and vital signs at admission. The principal indications for ICU admission were categorized as hypertensive disorders of pregnancy, obstetric hemorrhage, sepsis, cardiac disease, and other medical or obstetric conditions. Critical care interventions recorded included blood or blood-component transfusion, mechanical ventilation, vasopressor support, renal replacement therapy, and other supportive measures where documented. ICU length of stay and hospital length of stay were also recorded.

Delayed referral was defined as transfer of a critically ill obstetric patient to the tertiary care centre more than two hours after the decision to refer. The primary maternal outcome was mortality during the ICU or hospital stay. Maternal mortality was defined as death occurring during the index hospital admission among women included in the study. Secondary outcomes included recovery and discharge, ICU length of stay, hospital length of stay, requirement for advanced critical care interventions, and development of complications such as multi-organ dysfunction. The final maternal outcome was categorized as recovered and discharged or maternal death.

Sample size calculation

A complete enumeration sampling approach was used. All eligible obstetric ICU admissions during the study period were considered for inclusion. Of the 250 records screened, 126 did not meet the inclusion criteria, and four had incomplete records. Therefore, 120 eligible patients were included in the final analysis. No separate minimum sample-size calculation was used because the study included all eligible cases available during the predefined study period.

Statistical analysis

Data were entered and analyzed using IBM SPSS Statistics for Windows, version 25.0 (IBM Corporation, Armonk, USA). Continuous variables were summarized using mean ± standard deviation, while categorical variables were summarized as frequencies and percentages.

Categorical variables were compared between survivors and non-survivors using the Pearson chi-square test. Fisher's exact test was used when the expected cell frequencies were insufficient for the Pearson chi-square test. A two-sided p-value < 0.05 was considered statistically significant.

## Results

A total of 250 patient records were screened during the study period. Of these, 126 patients did not meet the inclusion criteria, and four had incomplete medical records. Therefore, 120 patients were included in the final analysis. The demographic and obstetric characteristics of the study population are presented in Table 1. The mean age of the patients was 26.8 ± 4.5 years. Most patients belonged to the 21-30-year age group (78, 65%), followed by those aged > 30 years (30, 25%) and < 20 years (12, 10%). Seventy-two patients (60%) were multigravida and 48 (40%) were primigravida. Regarding booking status, 42 patients (35%) were booked, and 78 (65%) were unbooked. At the time of ICU admission, 50 patients (41.7%) were antenatal, and 70 (58.3%) were postnatal.

**Table 1:** Demographic and Obstetric Characteristics (N = 120)

Variable	Value (%)
Age (years, Mean ± SD)	26.8 ± 4.5
Age Group (years)	
< 20	12 (10%)
21–30	78 (65%)
> 30	30 (25%)
Gravidity	
Primigravida	48 (40%)
Multigravida	72 (60%)
Booking Status	
Booked	42 (35%)
Unbooked	78 (65%)
Obstetric Status at Admission	
Antenatal	50 (41.7%)
Postnatal	70 (58.3%)

The indications for ICU admission are presented in Table 2. Hypertensive disorders of pregnancy, including preeclampsia and eclampsia, were the most frequently recorded indication, accounting for 42 (35.0%) admissions. Obstetric hemorrhage accounted for 36 (30.0%) admissions, while sepsis was reported in 18 (15.0%) patients. Cardiac disease accounted for 12 (10.0%) admissions, and other indications, including embolism and severe anemia, accounted for 12 (10.0%) admissions.

**Table 2:** Indications for Obstetric Intensive Care Unit Admission Among the Study Population (N = 120)

Indication	Number (%)
Hypertensive disorders (Preeclampsia/Eclampsia)	42 (35%)
Obstetric hemorrhage	36 (30%)
Sepsis	18 (15%)
Cardiac disease	12 (10%)
Others (embolism, severe anemia, etc.)	12 (10%)

The critical care interventions provided to the study population are presented in Table 3. Blood transfusion was the most frequently recorded intervention, provided to 84 (70.0%) patients. Mechanical ventilation was required in 48 (40.0%) patients, while vasopressor support was provided to 30 (25.0%) patients. Renal replacement therapy was required in 6 (5.0%) patients.

**Table 3:** Critical Care Interventions Provided to Obstetric ICU Patients (N = 120) RRT: renal replacement therapy.

Intervention	Number (%)
Blood transfusion	84 (70%)
Mechanical ventilation	48 (40%)
Vasopressor support	30 (25%)
Dialysis (RRT)	6 (5%)

Table 4 presents maternal outcomes. Of the 120 patients included in the study, 102 (85.0%) recovered and were discharged, while 18 (15.0%) died during the hospital admission.

**Table 4:** Maternal Outcomes (N = 120)

Outcome	Number (%)
Recovered and discharged	102 (85%)
Maternal mortality	18 (15%)

Table 5 compares selected clinical factors between survivors (N = 102) and non-survivors (N = 18). Delayed referral was documented in 30 (29.4%) survivors and 14 (77.8%) non-survivors, with a statistically significant difference between the groups (χ² = 15.41, p < 0.001). Multi-organ dysfunction was present in 12 (11.8%) survivors compared with 15 (83.3%) non-survivors, and this difference was statistically significant (χ² = 44.94, p < 0.001). Mechanical ventilation was required in 30 (29.4%) survivors and all 18 (100%) non-survivors, with a statistically significant difference between the groups (χ² = 31.76, p < 0.001). Delayed referral, multi-organ dysfunction, and requirement for mechanical ventilation were significantly associated with maternal mortality (all p < 0.001).

**Table 5:** Factors Associated with Maternal Mortality Categorical variables were compared between survivors and non-survivors using the Pearson chi-square (χ²) test. *p < 0.05 was considered statistically significant.

Variable	Survivors (N = 102)	Non-survivors (N = 18)	χ² value	p-value
Delayed referral	30 (29.4%)	14 (77.8%)	15.41	< 0.001*
Multi-organ dysfunction	12 (11.8%)	15 (83.3%)	44.94	< 0.001*
Mechanical ventilation	30 (29.4%)	18 (100%)	31.76	< 0.001*

## Discussion

This study assessed the demographic and clinical characteristics of obstetric patients admitted to the ICU, the indications for ICU admission, the critical care interventions provided, and maternal outcomes. The mean age of the patients was 26.8 ± 4.5 years, with the majority belonging to the 21-30-year age group. This finding is consistent with previous studies reporting that severe maternal morbidity and obstetric ICU admissions are commonly observed among women in the reproductive age group [13,14]. The predominance of younger women in the present study may reflect the age distribution of the obstetric population served by the study centre. However, age alone should not be considered an indicator of ICU outcome.

In the present study, 60% of the patients were multigravida and 65% were unbooked. The high proportion of unbooked women indicates the importance of access to regular antenatal care and timely identification of pregnancy-related complications. Previous studies have reported that inadequate antenatal care and socioeconomic barriers may contribute to delayed recognition and management of maternal complications, particularly in resource-limited settings [15]. The finding that 58.3% of ICU admissions occurred during the postnatal period also highlights the importance of continued monitoring after delivery, as serious maternal complications may develop or worsen during the postpartum period [16].

Hypertensive disorders of pregnancy were the most common indication for ICU admission in this study, accounting for 35% of admissions, followed by obstetric hemorrhage (30%). These findings are consistent with previous literature identifying hypertensive disorders and obstetric hemorrhage as major causes of severe maternal morbidity and obstetric ICU admission [17]. Hypertensive disorders may result in complications involving multiple organ systems, while obstetric hemorrhage can lead to hemodynamic instability and the need for urgent blood component therapy. The presence of sepsis in 15% of admissions and cardiac disease in 10% further demonstrates the contribution of both obstetric and medical conditions to critical maternal illness. Early recognition, appropriate stabilization, and timely referral remain important components of management in these conditions [10].

The critical care interventions observed in this study reflected the severity and clinical requirements of the admitted patients. Blood transfusion was required in 70% of patients, followed by mechanical ventilation in 40%, vasopressor support in 25%, and renal replacement therapy in 5%. The high requirement for blood transfusion is consistent with the substantial proportion of ICU admissions due to obstetric hemorrhage. Mechanical ventilation, vasopressor support, and renal replacement therapy are generally required in patients with severe respiratory, cardiovascular, or renal compromise. Similar use of advanced organ-support interventions has been reported in studies of critically ill obstetric patients [18]. Because several interventions could be provided to the same patient, these percentages should be interpreted as the proportion of patients receiving each intervention rather than mutually exclusive categories.

In this study, 102 (85%) patients recovered and were discharged, whereas 18 (15%) patients died. The observed mortality represents the outcome of a group of women who had already developed sufficiently severe illness to require ICU admission and should therefore be interpreted within the context of the severity of the study population. Differences in ICU mortality between settings may be influenced by case mix, referral patterns, availability of critical care resources, timing of presentation, and access to multidisciplinary care [12]. Therefore, the present findings should not be interpreted as a direct measure of ICU effectiveness or as evidence of treatment efficacy.

The comparison between survivors and non-survivors demonstrated significant associations between delayed referral, multi-organ dysfunction, mechanical ventilation, and maternal mortality. Delayed referral was more frequent among non-survivors than survivors, suggesting an association between delay in reaching higher-level care and adverse maternal outcome. This finding is consistent with the three-delays framework, in which delays in seeking care, reaching an appropriate facility, and receiving appropriate care may contribute to poor maternal outcomes [18]. However, the present study evaluated referral delay as an observed clinical factor and did not assess the individual components of the three-delays model separately.

Multi-organ dysfunction was also significantly more frequent among non-survivors than survivors. This finding is clinically plausible because dysfunction involving multiple organ systems reflects severe systemic illness. However, the present analysis used categorical comparison with the chi-square test and therefore demonstrates an association rather than an independent predictive effect. Importantly, some patients with multi-organ dysfunction survived, and therefore MOD should not be described as a predictor of mortality based on the present analysis alone.

Similarly, mechanical ventilation was significantly associated with maternal mortality. All non-survivors in the present dataset required mechanical ventilation, whereas mechanical ventilation was also required in a proportion of survivors. Mechanical ventilation in this context is more appropriately considered a marker of severe illness and the need for advanced organ support rather than an independent cause or predictor of mortality. The relationship between mechanical ventilation, multi-organ dysfunction, delayed referral, and mortality may also reflect the underlying severity of illness.

The findings regarding delayed referral, multi-organ dysfunction, and mechanical ventilation should therefore be interpreted as unadjusted associations. The present study used chi-square analysis to compare selected categorical variables between survivors and non-survivors. Multivariable analysis would be required to determine whether these factors are independently associated with maternal mortality after accounting for potential confounding and interrelationships between measures of disease severity. This limitation is particularly relevant because delayed referral, multi-organ dysfunction, and mechanical ventilation may occur together in critically ill patients.

The findings also emphasize the importance of timely recognition and referral of women with potentially life-threatening obstetric complications. Strengthening antenatal care, improving referral pathways, ensuring timely access to blood and blood components, and maintaining availability of multidisciplinary critical care services may help healthcare facilities manage severe maternal complications more effectively [10,12]. These measures are particularly relevant in referral centres serving populations from areas with variable access to emergency obstetric and critical care services.

Strengths and limitations

A strength of this study was the inclusion of all eligible obstetric ICU admissions during the predefined study period rather than selecting a predetermined sample. The study also assessed demographic characteristics, indications for ICU admission, critical care interventions, and maternal outcomes within the same cohort. The inclusion of clinically relevant factors such as delayed referral, multi-organ dysfunction, and mechanical ventilation provided additional information regarding factors associated with mortality.

This study had several limitations. First, it was conducted at a single tertiary care centre, which may limit the generalizability of the findings to other hospitals or healthcare settings. Second, the sample size was relatively small, with 120 patients included in the final analysis. Third, the retrospective design depended on the completeness and accuracy of routinely recorded medical information. Fourth, the study did not include long-term maternal follow-up after hospital discharge. Finally, the analysis of factors associated with mortality was based on unadjusted categorical comparisons; therefore, the observed associations should not be interpreted as independent predictors or causal relationships.

## Conclusions

Hypertensive disorders and obstetric hemorrhage were the leading indications for obstetric ICU admission in this study. Delayed referral, multi-organ dysfunction, and requirement for mechanical ventilation were significantly associated with maternal mortality. These findings highlight the importance of timely recognition and referral of critically ill obstetric patients and the availability of multidisciplinary critical care services. However, the observed associations should be interpreted in the context of the single-centre retrospective design and should not be considered independent predictors of mortality.
